# Hyperaccumulator *Solanum nigrum* L. Intercropping Reduced Rice Cadmium Uptake under a High-Bed and Low-Ditch Planting System

**DOI:** 10.3390/plants12234027

**Published:** 2023-11-30

**Authors:** Rakhwe Kama, Qingguang Ma, Farhan Nabi, Maimouna Aidara, Peiyi Huang, Zhencheng Li, Juxi He, Sekouna Diatta, Huashou Li

**Affiliations:** 1 Guangdong Provincial Key Laboratory of Agricultural & Rural Pollution Abatement and Environmental Safety, College of Natural Resources and Environment, South China Agricultural University, Guangzhou 510642, China; krakhwe@yahoo.fr (R.K.); mqg463367252@163.com (Q.M.); khosofarhan23@gmail.com (F.N.); 20223138028@stu.scau.edu.cn (P.H.); lizhencheng@stu.scau.edu.cn (Z.L.); 15918844926@163.com (J.H.); 2 Guangdong Provincial Key Laboratory of Utilization and Conservation of Food and Medicinal Resources in Northern Region, Shaoguan University, Shaoguan 512005, China; 3 Laboratory of Ecology, Faculty of Sciences and Technology, Cheikh Anta University of Dakar, Dakar 50005, Senegal; mounasadou@gmail.com (M.A.); sekouna.diatta@ucad.edu.sn (S.D.)

**Keywords:** Cd, high-bed and low-ditch planting system, hyperaccumulator, intercropping system, *Solanum nigrum* L.

## Abstract

Anthropogenic activities have raised cadmium (Cd) concentrations in agricultural soil, emerging as a primary catalyst for the decline in crop yield. Intercropping of two or several plants is one technique among many Cd phytoremediation techniques that has gained enormous attention recently. However, the impact of cultivation modes on Cd movement in rice plants when intercropped with heavy metal (HM) hyperaccumulator plants remains unclear. Thus, this study was designed to explore the effects of cultivation modes and the intercropping of rice with *Solanum nigrum* L. on rice growth and Cd uptake in Cd-contaminated soil. The experimental design encompassed five treatments: dry cultivation of monocultured rice, monocultured *Solanum nigrum* L., and intercropped rice–*Solanum nigrum* L.; flood cultivation of monocultured rice; and intercropped rice–*Solanum nigrum* L. in a high-bed and low-ditch planting system. The results revealed a significant increase in rice growth when intercropped with *Solanum nigrum* L., with a notable increase of 18.32 g∙plant^−1^ observed in rice biomass in dry cultivation under the intercropping system. In contrast, a more modest increase of 3.67 g∙plant^−1^ was observed in the high-bed and low-ditch intercropped rice–*Solanum nigrum* L. mode. The soil total Cd was higher in dry cultivation of monocultured rice and *Solanum nigrum* L. compared to intercropped rice/*Solanum nigrum* L.-cultivated soil, with lower values recorded for intercropped rice/*Solanum nigrum* L. under the high-bed and low-ditch planting system. In contrast, no significant effect was noted on soil exchangeable Cd content based on the planting pattern and cultivation mode. Intercropping with *Solanum nigrum* L. demonstrated a significant reduction of Cd content in various rice tissues, particularly in roots at the maturity stage, while Cd content was reduced across all rice tissues under the high-bed and low-ditch planting system. The Cd content in the stem, leaves, and bran of monocropped rice was higher compared to intercropped rice. This study suggests that the rice–*Solanum nigrum* L. intercropping system effectively reduces rice Cd uptake, particularly under the high-bed and low-ditch planting system.

## 1. Introduction

Soil is a crucial natural element and a valuable agricultural resource essential for human survival. It is the foundation of the entire food chain, providing essential nutrients and support for crop growth. The absence of healthy soil would severely curtail our ability to produce food, which would increase the challenges in feeding the world’s population. Heavy metal (HM) contamination of soil is a prominent environmental problem that humanity is currently facing and has attracted much attention from both industry and the general public [1,2,3,4]. Soil HM contamination is mainly due to the diffusion, deposition, and accumulation of HMs in industrial solid waste as well as agricultural land irrigated with wastewater containing HMs [2,3,5]. Additionally, the large-scale application of HM-containing pesticides and phosphorus fertilizers significantly contributes to this predicament [3]. HMs such as cadmium (Cd), lead (Pb), and arsenic (As) cause serious environmental and health issues [3]. For instance, Cd, which is common in food pollution, constitutes a serious threat to human health. Cd is not an essential element for plant nutrition; moreover, it is highly toxic and hinders plant growth.

Currently, there are three primary methods [6] for HM-contaminated soil remediation: physical, chemical, and biological remediation [2,5,7]. The main idea of bioremediation, a subset of which is phytoremediation, is that it harnesses biological organisms’ metabolic processes to transfer, decompose, or contain harmful substances, thereby reducing the concentration of pollutants in the surrounding environment [8]. Phytoremediation is an extraction, absorption, decomposition, transformation, or immobilization method of HMs using plants to reduce and eliminate soil HM pollution [8,9]. For instance, phytoextraction is a method of transferring HMs from the soil to the roots and aerial plant parts, using HM-hyperaccumulating plants to absorb HMs and reduce their concentrations in the soil [10].

Phytoremediation is considered one of the most advantageous remediation techniques due to its cost-effectiveness, suitability for large-scale application, ecological benefits, and environmental friendliness [10]. Distinctive plant species known as accumulators and hyperaccumulators have the ability to absorb and accumulate high levels of HMs in their tissues without being negatively affected [11,12]. These plants have shown promising results in removing HMs such as Cd, Zn, and Pb from contaminated soil [12]. Many HM hyperaccumulators, including *Solanum nigrum* L., have been discovered in China [13]. *Solanum nigrum* L. plant, known as a Cd hyperaccumulator, is native to Eurasia and was recently discovered in China [9,14]. Research on *Solanum nigrum* L. as an HM extractor was carried out by Khalid et al. in 2019 [15]. *Solanum nigrum* L. typically grows as a weed in different environments. It can also be grown in tropical and subtropical agroclimatic zones [13]. It has been found that the remediation efficiency of *Solanum nigrum* L. is relatively high in low Cd-contaminated soils, while it is significantly lower in high Cd-contamination conditions [14]. *Solanum nigrum* L. proves adept at effectively absorbing and accumulating Cd in the soil, concurrently expediting the synthesized metallothionein to chelate Cd ions to alleviate and weaken the damage of Cd to itself [9,16,17]. In maize intercropping systems, *Solanum nigrum* L. exhibits higher Cd uptake and translocation capacities than other hyperaccumulators, making it an ideal phytoremediation material in such systems [13,14]. However, further studies are still needed to determine the effects of *Solanum nigrum* L. on rice Cd uptake under different cultivation modes. 

In comparison to monocropping, the intercropping system is conducive to field ventilation and light penetration, prevention, and pest and disease control while increasing the accumulation of organic matter and improving yields [1,5,7,12]. Intercropping is a well-established agricultural practice that substantially contributes to the sustainable development of agriculture [18,19]. Concerning the high-bed and deep furrow cultivation mode, it has been suggested that the furrow elevation is beneficial for the growth of dryland vegetation and waterlogged vegetation, respectively [20,21]. In addition, this cultivation mode effectively reduces water loss, soil erosion, and organic matter decomposition [21]. However, the effects of the intercropping system on rice Cd uptake under the high-bed and low-ditch cultivation mode require further investigations. 

Rice is a staple crop in South China [22] that faces challenges from Cd contamination in paddy fields, which is affecting the quality and safety of rice and induces health risks to human beings [5,23]. Thus, the issue of soil Cd contamination has gained wide public attention, and alternatives should be found to remediate this issue without reducing rice production. The paddy field ecosystem, an artificial ecosystem comprised of soil and rice, is characterized by flooding effects, frequent agricultural activity, and the ability of rice to accumulate Cd [10,23]. Consequently, Cd contamination in farmland and rice planting has become a serious concern that needs to be tackled [23]. Intercropping rice with wetland plants has been proven to significantly reduce Cd content in rice while increasing yield [24,25]. However, there are very few aquatic plants that can be used for phytoremediation, and their biomasses are small. The Cd hyperaccumulator plants found so far are all arid plants that are not able to grow under flooding conditions. Thus, there is a necessity to explore Cd hyperaccumulator plants with more substantial biomasses conducive to intercropping with rice with a better restoration effect and suitable planting. Exploring suitable hyperaccumulator plants with higher biomass and better restoration effects for intercropping with rice and their appropriate planting requirements have become the key to the application prospect of rice intercropping restoration in paddy fields. Therefore, this study attempted to explore the effect of cultivation mode and intercropping system on Cd-contaminated soil remediation using *Solanum nigrum* L. and rice as plant materials and properly changing water conditions, as well as creating suitable environmental conditions for the common growth of rice and hyperaccumulator plants. Inspired by the base pond system and the raised-bed and deep furrow planting pattern common in the low-lying waterlogged areas of the Pearl River Delta [21], we proposed the intercropping pattern of *Solanum nigrum* L.-rice using a high bed and low ditch to investigate the remediation effect and discuss the feasibility of applying the pattern.

## 2. Results

### 2.1. Variation in Rice Growth Parameters

Table 1 shows the variation of different rice plant part biomasses under different cultivation modes. The results showed that root dry weight was higher under the monocropping system compared to the intercropping system in dry cultivation mode. Conversely, in dry cultivation, the dry weight of rice stems and leaves demonstrated a substantial increase of 18.32 g∙plant^−1^ and 8.67 g∙plant^−1^, respectively, under the intercropping system compared with the monocropping system. This study suggests that the intercropping system increases rice aboveground biomass, with a 157.39% and 146.70% increase for stems and leaves, respectively, in dry cultivation.

Rice growth parameters were higher in intercropped rice under the high-bed and low-ditch planting system compared with monocultured rice in flood cultivation, except for root dry weight. Nevertheless, this increase was not as obvious as in dry cultivation conditions. These findings indicate that the intercropping system increases rice growth parameters.

### 2.2. Total Cd and Exchangeable Cd Content in Soil under Different Cultivation Modes

**Figure 1:**
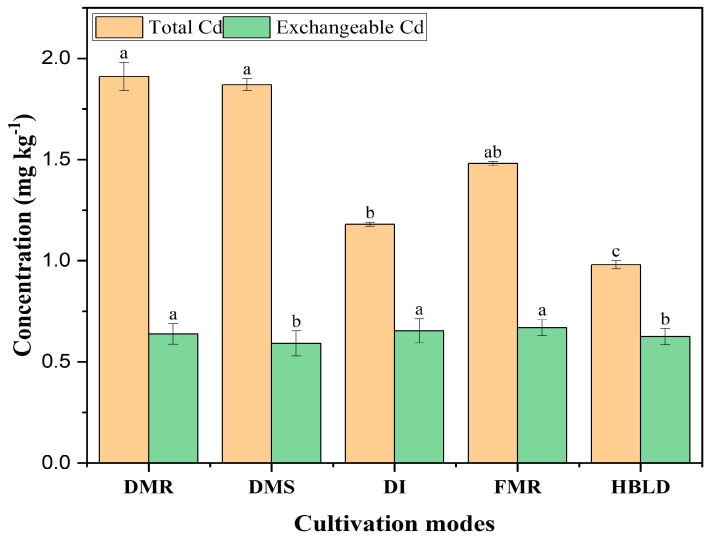
Total Cd and exchangeable Cd concentration in soil under different cultivation modes. Note: DMR refers to dry cultivation of monocultured rice; DMS refers to dry cultivation of monocultured *Solanum nigrum* L.; DI refers to dry cultivation of intercropped rice and *Solanum nigrum* L.; FMR refers to flood cultivation of monocultured rice; HBLD refers to high-bed and low-ditch cultivation of intercropped rice and *Solanum nigrum* L. The values represent the means (±standard error) of data obtained in the experiment (*n* = 3); different letters in the same column indicate significant differences between treatments (*p* < 0.05).

Figure 1 shows the variation of Cd content in soil at the end of the experiment. Significant differences were observed in Cd concentration based on the cultivation mode and planting pattern. Higher concentrations were recorded for monocultured rice and *Solanum nigrum* L. in dry and flood cultivation modes compared with intercropped rice in dry and high-bed and low-ditch cultivation modes. The concentration of Cd in soil was significantly affected by the cultivation mode, with a significant decrease under the high-bed and low-ditch system (Figure 1). In addition to the cultivation mode, the planting pattern also had significant effects on Cd concentration in soil, with lower values recorded under the intercropping system. This study suggests that the intercropping system decreases soil Cd content with more pronounced effects under the high-bed and low-ditch planting system. 

Concerning the variation in exchangeable Cd concentration under different treatments, a slight increase was noted under the intercropping system compared to the monocropping system in dry cultivation (Figure 1). A different situation was observed in intercropped rice and *Solanum nigrum* L. in high-bed and low-ditch soil compared to monocropped rice under flooded soil in which higher values were recorded. 

### 2.3. Inter-Root Soil pH Variation under Different Cultivation Modes

Table 2 shows the significant effects of cultivation modes on inter-root soil pH. A significant increase in soil pH was observed in all cultivation modes compared with the original soil pH (4.11). Higher pH values were observed under the intercropping system compared to the monocropping system. In addition, the soil pH was greater under the high-bed and low-ditch mode, as well as flood cultivation modes, compared with the dry cultivation mode. Moreover, the inter-root soil pH was higher under intercropped *Solanum nigrum* L.-cultivated soil compared with other treatments. This study suggests that plant–soil interactions increase soil pH with a more significant impact in flood and high-bed and low-ditch cultivation modes.

### 2.4. Cd Content in Various Parts of Rice under Different Cultivation Modes

**Figure 2:**
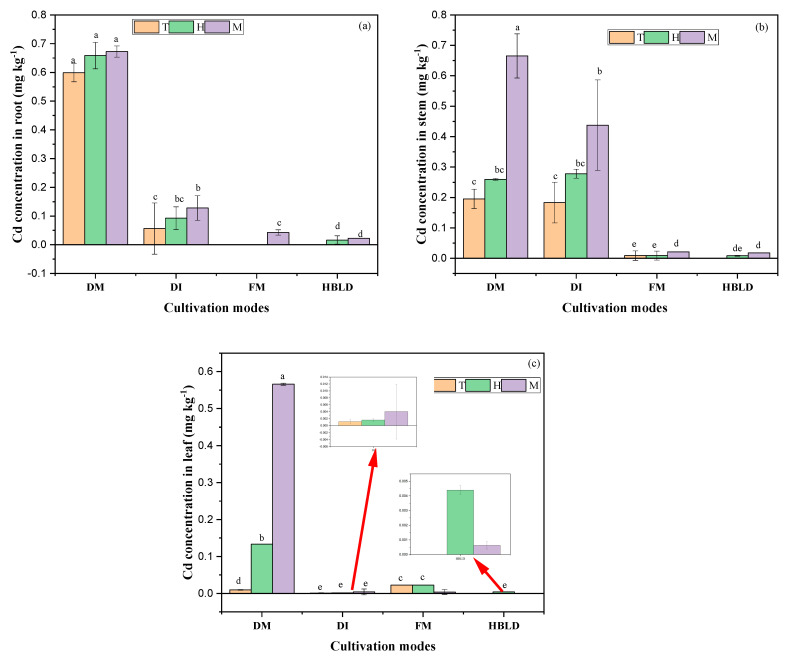
Cd content in rice roots (**a**), stem (**b**), and leaf (**c**) under different cultivation modes. Note: DM refers to dry cultivation of monocultured rice; DI refers to dry cultivation of intercropped rice; FM refers to flood cultivation of monocultured rice; HBLD refers to high-bed and low-ditch cultivation of intercropped rice. T means tillering stage; H means heading stage; M means maturity stage. The values represent the means (±standard error) of data obtained in the experiment (*n* = 3); different letters in the same column indicate significant differences between treatments (*p* < 0.05).

Changes in Cd concentration in various parts of rice under different cultivation modes and planting patterns are shown in Figure 2. The results showed higher Cd content in the roots of monocropped rice during the tillering and heading phases compared with intercropped rice in dry cultivation mode. However, higher Cd content was observed in intercropped rice in dry cultivation mode at the maturity stage. A different situation was observed in monocultured rice in flood cultivation and intercropped rice in a high-bed and low-ditch planting system, where Cd was detected only from the heading stage for the high-bed and low-ditch planting system and from the maturity stage for flood cultivation. 

As shown in Figure 2b, the Cd content in rice stems under intercropping treatment was lower compared to monocropped rice stems at the tillering stage. A similar trend was observed between monocultured rice under flood-cultivated and intercropped rice in the high-bed and low-ditch system, where Cd content was significantly higher in monocultured rice stems. It is also important to mention that rice Cd content in stems was lower under flood and high-bed and low-ditch cultivation modes compared to dry cultivation. 

Concerning the concentration of Cd in rice leaves, higher values were noted under monocultured rice in dry cultivation compared with other treatments (Figure 2c). This study demonstrated that in addition to the intercropping system, flood cultivation and high-bed and low-ditch planting systems reduce rice Cd uptake, with more pronounced effects observed under the high-bed and low-ditch planting system.

### 2.5. Cd Content in Rice Grain and Bran under Different Cultivation Modes

**Figure 3:**
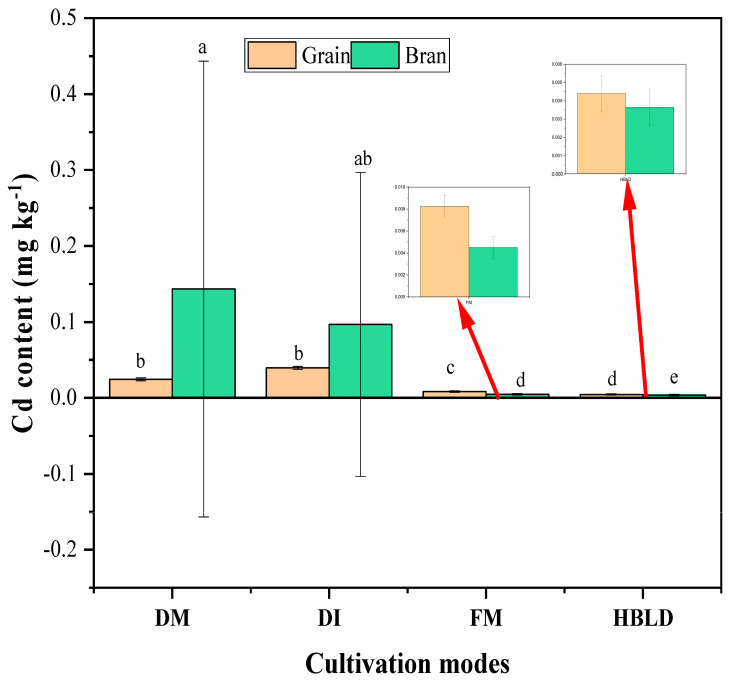
Cd content in rice grain and bran under different cultivation modes. Note: DM refers to dry cultivation of monocultured rice; DI refers to dry cultivation of intercropped rice; FM refers to flood cultivation of monocultured rice; HBLD refers to high-bed and low-ditch cultivation of intercropped rice. The values represent the means (±standard error) of data obtained in the experiment (*n* = 3); different letters in the same column indicate significant differences between treatments (*p* < 0.05).

As shown in Figure 3, Cd content in rice grain under the intercropping system was lower than that of the monocropping system in dry cultivation mode, contrastingly to the rice bran, in which higher Cd content was noted under an intercropping system in dry cultivation mode. Concerning the flood monocropped rice and intercropped rice in high-bed and low-ditch cultivation modes, the content of Cd in bran and grain was lower in high beds and low ditches than that of the single-crop flooding mode. 

### 2.6. Cd Content in Solanum nigrum L. under Different Cultivation Modes

**Figure 4:**
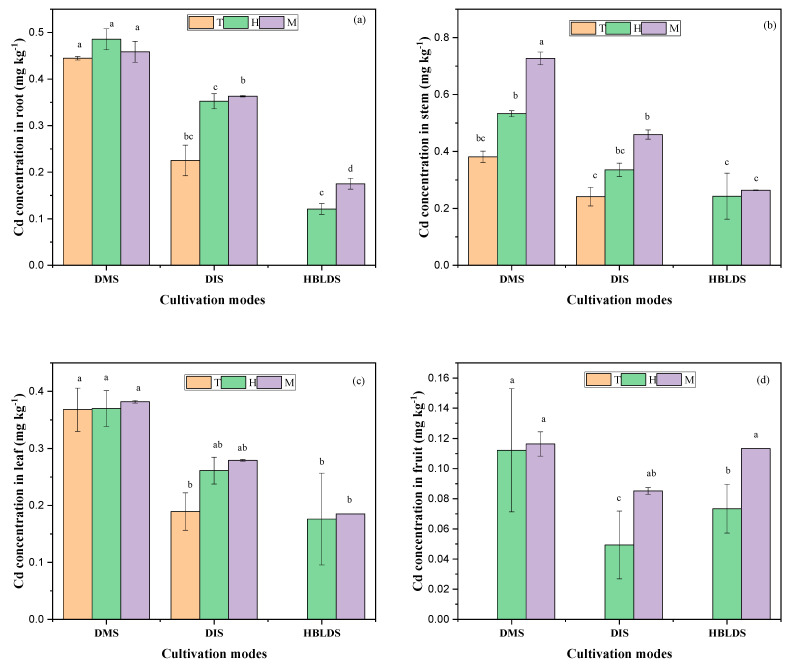
Cd content in *Solanum nigrum* roots (**a**), stem (**b**), leaf (**c**), and fruit (**d**) under different cultivation modes. Note: DMS refers to dry cultivation of monocultured *Solanum nigrum* L.; DIS refers to dry cultivation of intercropped *Solanum nigrum* L.; HBLDS refers to high-bed and low-ditch cultivation of intercropped *Solanum nigrum* L. T means tillering stage; H means heading stage; M means maturity stage. The values represent the means (±standard error) of data obtained in the experiment (*n* = 3); different letters in the same column indicate significant differences between treatments (*p* < 0.05).

The changes in Cd content in various parts of both monocropped and intercropped *Solanum nigrum* L. under different cultivation modes and planting patterns are shown in Figure 4. The results show that Cd content in *Solanum nigrum* L. was lower under the intercropping system compared to the monocropping system. In addition, significant differences in Cd content were noted at different phases. Higher Cd content was noted in *Solanum nigrum* L. roots compared to other plant parts in dry cultivation mode, except for leaves at the tillering stage. Concerning the high-bed and low-ditch cultivation mode, higher Cd content was noted in leaves and fruits compared to other parts (Figure 4). However, higher values were recorded during the heading phase in roots and stems, contrastingly to leaves, in which higher values were recorded in the maturity stage and heading stage.

### 2.7. Accumulation and Dynamic of Cd in Soil–Rice System under Different Cultivation Modes

**Figure 5:**
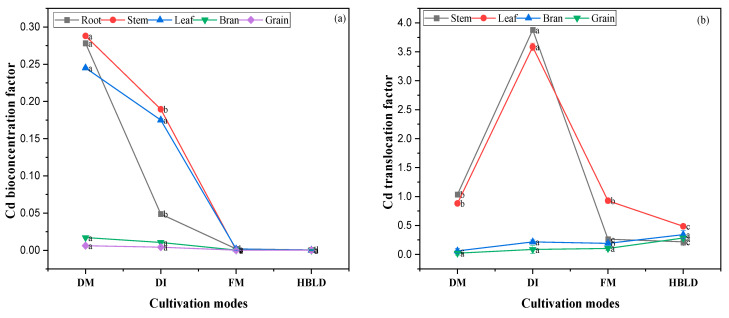
Cd BCF (**a**) and TF (**b**) in rice under different cultivation modes. Note: DM refers to dry cultivation of monocultured rice; DI refers to dry cultivation of intercropped rice; FM refers to flood cultivation of monocultured rice; HBLD refers to high-bed and low-ditch cultivation of intercropped rice. The values represent the means (±standard error) of data obtained in the experiment (*n* = 3); different letters in the same column indicate significant differences between treatments (*p* < 0.05).

Significant changes were noted regarding Cd bioconcentration factor (BCF) and translocation factor (TF) across all treatments. The planting patterns and cultivation modes had significant effects on BCF (Figure 5a). High BFC was noted in monocropped rice, followed by intercropped rice in dry cultivation. A slight increase of BCF was noted in intercropped rice under the high-bed and low-ditch planting system in comparison with monocultured rice in flood cultivation. A similar trend was observed concerning the TF of Cd in various parts of rice except under dry cultivation, where the TF was higher under the intercropping system compared to the monocropping system (Figure 5b). This study shows that the high-bed and low-ditch planting system, as well as the flood cultivation mode, decrease Cd BCF. In addition, it was also found that the intercropping system increases Cd TF in various parts of rice.

## 3. Discussion

### 3.1. Effects of Intercropping and Cultivation Mode on Rice Growth

Cultivation mode plays a crucial role in rice production. The effects of cultivation modes on rice growth have been previously investigated [20,21]. For instance, it has been found that a dry cultivation mode increases root oxidation activity, photosynthesis, and, therefore, rice growth [26]. In addition, it has also been revealed that a dry cultivation mode enhances key enzyme activities [21,27]. This study aligns with previous research suggesting that a dry cultivation mode increases rice growth parameters with enhanced effects under the intercropping system [18,19]. Moreover, the high-bed and low-ditch cultivation mode increases rice growth compared to other cultivation modes [20,21]. This situation could be explained by the fact that rice is less exposed to potential toxic heavy metals in soil and water, which could hinder its growth. This study is consistent with previous studies suggesting that the dry cultivation mode can facilitate rice growth, decrease evapotranspiration, and increase water use efficiency. 

### 3.2. Effects of Intercropping System and Cultivation Mode on Rice Cd Uptake

The intercropping system is a farming practice that enables crops to share the available resources and complement each other [1]. It can also affect heavy metal uptake by plants due to the alteration of the rhizosphere microorganism community and root exudation [7]. The results showed a significant decrease in rice Cd uptake under the intercropping system compared with the monocropping system, especially under the high-bed and low-ditch cultivation mode. A significant decrease in rice Cd uptake was also observed in flood cultivation mode, which might be due to the significant amount of Cd that can be accumulated in water. This study supports previous studies suggesting that the intercropping system decreases plant HM uptake [1,5,11]. In addition, it is important to mention that less Cd was accumulated in rice grown under the high-bed and low-ditch cultivation mode [28]. This situation could be explained by the position of rice plants, which were not very exposed to the contaminated soil, as well as the presence of the hyperaccumulator *Solanum nigrum* L. In line with previous studies, this study demonstrated that the intercropping system decreases rice Cd uptake. 

### 3.3. Effects of Intercropping and Cultivation Mode on Inter-Root Soil pH

This study highlights the significant impacts of the plant–soil interaction on soil pH across all treatments. A significant increase in soil pH was observed under cultivated soil in comparison with the original soil, with higher values recorded under the intercropping system and in the dry cultivation mode, as well as in the high-bed and low-ditch planting system (Table 2) [28]. This study is consistent with previous studies that showed that rhizosphere-mediated processes are strong predictors of soil chemical properties [29]). This increase in soil pH, which was higher under an intercropping system in the dry cultivation mode, indicates synergetic actions between rice and *Solanum nigrum* to improve soil health and resource use efficiency. 

### 3.4. Effects of Intercropping and Cultivation Mode on Inter-Root Soil Exchangeable Cd

Figure 1 shows that the Cd exchangeable content of rice inter-root soil in high-bed, low-ditch planting was significantly lower than in other cultivation modes [24,28]. However, the Cd exchangeable content of each inter-root soil after the experiment was significantly higher than the background value, suggesting positive impacts concerning the phytoremediation process. Therefore, the high-bed and low-ditch and flood cultivation modes, as well as the intercropping system under a dry cultivation mode, could be considered serious options for the phytoremediation process of rice-cultivated and contaminated soil. It is well known that flooded conditions increased the ability of soil organic matter to complex Cd, which led to a relatively stable accumulation of Cd near the inter-root soil, resulting in an increase in inter-root soil Cd exchangeable content, which in turn led to an increase in the Cd content in the roots of rice [20]. This study showed that the high-bed and low-ditch planting system decreases soil exchangeable Cd content.

### 3.5. Effects of Intercropping and Cultivation Mode on Cd Accumulation and Mobility in Soil Plant System

The BCF is defined as the ability of a plant to accumulate HMs from the soil into its various parts [30]. It is the ratio of the concentration of HMs in plants to the concentration in the corresponding soil [31]. Higher BCF was observed in dry cultivation mode, which might be due to the continuous contact between the rice plant and the contaminated soil. Contrastingly to the dry cultivation mode, lower BCFs were noted in various parts of rice in the flood cultivation and high-bed and low-ditch planting systems. This situation might be explained by the potential accumulation of Cd in water under flood cultivation and the lack of significant contact between rice plants and the contaminated soil under the high-bed and low-ditch cultivation mode. This study is in line with previous studies suggesting that intercropping increases HM TF [1,5,32]. This study aligns with previous studies suggesting that the use of hyperaccumulators such as *Solanum nigrum* L. reduces HM concentration in soil [13,15]. This study showed that *Solanum nigrum* L. played a significant role in reducing Cd concentration in soil and uptake in rice plants. However, it is important to mention that there should be a follow-up to remove the hyperaccumulator plants from the soil after death to prevent the accumulated Cd from returning to the soil.

## 4. Materials and Methods

### 4.1. Site Description and Experimental Materials

The experiment was conducted in ecological farm of Natural Resources and Environment College, South China Agricultural University, Guangzhou City, China. The soil used was collected from the central area of farmland in Xinjiang Town, Wengyuan, downstream of Dabaoshan Mining area, Guangdong Province (24°29°31″ N; 113°48°39″ E). The rice plant used in this study is a low-accumulation rice variety, Zhueryou 918, and the Cd hyperaccumulator plant was *Solanum nigrum* L. The low-accumulation rice variety was selected to promote the accumulation of Cd towards the hyperaccumulator *Solanum nigrum* L. The basic chemical properties of the used Cd-contaminated soil are as follows: organic matter (OM): 30.17 g kg^−1^; total nitrogen: 1.98 g kg^−1^; available phosphorus content: 51.30 mg kg^−1^; available potassium: 45.20 mg kg^−1^; available Cd: 0.27 mg kg^−1^; and total Cd concentration: 2.31 mg kg^−1^.

### 4.2. Experimental Design

**Figure 6:**
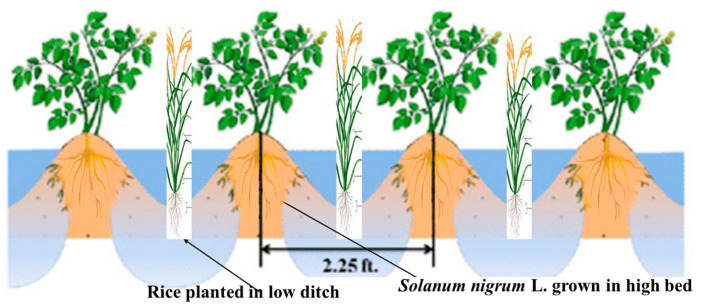
Scheme illustrating high-bed and low-ditch planting system.

The experiment was conducted with a total of five treatments, dry cultivation of monocultured rice, monocultured *Solanum nigrum* L., intercropped rice–*Solanum nigrum* L., flood cultivation of monocultured rice, and intercropped rice–*Solanum nigrum* L., in high beds and low ditches. However, the main objective of this study was focused on rice Cd uptake. The flooding experiment was carried out in a greenhouse with three replications. The planting scheme for the intercropped rice–*Solanum nigrum* L. treatment involved two *Solanum nigrum* L. plants on high beds while maintaining the surrounding soil slightly moist, along with two rice plants grown under flooded conditions within the furrows (Figure 6). Additionally, four monocropped rice plants were cultivated under flooded conditions. The dry cultivation experiment was conducted outdoors with 3 replicates and a total of 9 pots, including monocropped *Solanum nigrum* L., monocropped rice, and intercropped *Solanum nigrum* L.-rice. The number of *Solanum nigrum* L. and rice in monocropping system was 6, while the ratio of rice: *Solanum nigrum* L. under intercropping system was 3:3.

### 4.3. Sampling Methods and Sample Conservation

#### 4.3.1. Plant Sampling and Preservation Methods

Plants grown in dry cultivation mode were sampled at rice tillering, heading, and maturity stages, whereas monocultured rice in flood cultivation and intercropped rice–*Solanum nigrum* L., in a high-bed and low-ditch planting system, were sampled at the heading and maturity stages. Rice and *Solanum nigrum* L. samples were taken with their respective roots, washed, and divided into parts. Rice was divided into roots, stems, leaves, rice grains, and bran at maturity, while *Solanum nigrum* L. was divided into roots, stems, leaves, and fruits. The samples were then subjected to an initial heating at 105 °C for 30 min to halt photosynthetic activity, followed by drying at 40 °C for approximately 3 to 5 days. Subsequently, the dried samples were weighed, crushed, and placed in sealed plastic bags for preservation.

#### 4.3.2. Soil Sample Sampling and Preservation Methods

Soil samples were collected at the maturity stage from 0 to 10 cm depth near the roots of both rice and *Solanum nigrum* L. plants using a soil extractor. The collected soil samples were naturally dried, ground, sieved through a 2 mm sieve, and stored in sealed bags.

### 4.4. Measurement Methods

#### 4.4.1. Determination of Cd Content in Various Parts of Rice and *Solanum nigrum* L.

A total of 0.10 g of plant sample was weighed and placed in a disintegration tube with the addition of 10 mL of nitric acid. The tube was then preheated at 120 °C for 20 min in an acid catcher and transferred to the disintegrator. After disintegration, the tube was placed in the acid catcher at 180 °C for post-disintegration acid catching. After disintegration, the final solution was then poured into a 25 mL volumetric flask, filtered, and stored after adjusting its volume with ultrapure water. The Cd content was then determined using the graphite furnace atomic absorption method. This process was followed for each plant part.

#### 4.4.2. Determination of Soil pH

A total of 10 g of soil sample was weighed and placed in a 50 mL centrifuge tube in which 20 mL of deionized water was added. The tube was shaken for 3–5 min (at 200 rpm/min) before its pH value was determined with a pH meter after 30 min of standing.

#### 4.4.3. Determination of Soil Exchangeable Cd Content

A total of 2 g of soil sample (through 60 mesh) was placed in a 50 mL plastic centrifuge tube. Subsequently, 16 mL of 1 mol/L MgCl^2^ solution was added. The sample was vibrated on a shaker for 2 h at a temperature of 20 ± 3 °C, centrifuged for 30 min (at a rotational speed of 4000 rpm/min), and filtered before collecting the supernatant. Exchangeable Cd content was measured using flame atomic absorption spectrometry (ZEEnit 700P, FAAS, Jena, Germany).

### 4.5. Statistical Analysis

Cd bioconcentration and translocation factors were calculated using the following equations [33].

(1) $$Bioconcentration\;factor\;(BCF)=\frac{Cd\;concentration\;in\;a\;plant\;part}{initial\;soil\;Cd\;total\;concentration}$$

(2) $$Translocation\;factor\;(TF)=\frac{Cd\;concentration\;in\;an\;aerial\;part}{root\;Cd\;concentration}$$

The experimental data were processed using Excel software (version 2019) and Origin Pro 8, and the statistical analysis was conducted using multifactor (univariate) analysis of variance (ANOVA) in SPSS 20.0. Duncan’s test (*p* < 0.05) was used to compare the significance of the differences between treatments.

## 5. Conclusions

This study showed that an intercropping system with dry cultivation, a high-bed and low-ditch planting system, and a monocropping system with flood cultivation decrease rice Cd uptake. The overall rice Cd uptake was lower in the high-bed and low-ditch and flood cultivation modes than in the dry cultivation mode. In addition, the concentration of Cd in rice grains was under permissible limits for human consumption. This study suggests that the high-bed and low-ditch planting system could be considered a serious option for phytoremediation of rice-cultivated and HM-contaminated soil. However, further studies are needed to determine the long-term feasibility and its impacts on the ecological consequences of the cultivated zone.

## Figures and Tables

**Table 1:** Effect of cultivation modes on rice biomass (g∙plant^−1^).

Cultivation Mode	Rice Dry Weight		
	RDW	SDW	LDW
dry intercropping	2.02 ± 0.34 ab	29.96 ± 11.37 a	14.58 ± 2.11 a
dry monoculture	3.89 ± 0.15 a	11.64 ± 4.86 b	5.91 ± 0.88 bc
intercropping high-bed and low-ditch planting system	1.05 ± 0.14 c	11.39 ± 0.55 b	6.06 ± 0.86 b
flooding monoculture	1.11 ± 0.27 b	7.72 ± 0.90 c	4.38 ± 0.27 c

Note: RDW refers to root dry weight; SDW refers to shoot dry weight; LDW refers to leaves dry weight. The values represent the means (±standard error) of data obtained in the experiment (*n* = 3); different letters in the same column indicate significant differences between treatments (*p* < 0.05).

**Table 2:** Effects of cultivation on pH value of rhizosphere soil of each crop.

Cultivation Mode	Plants	Treatments	pH
Dry cultivation	rice	dry intercropping	4.49 ± 0.04 ab
		dry monoculture	4.31 ± 0.08 ab
	*Solanum nigrum* L.	dry intercropping	4.52 ± 0.05 a
		dry monocropping	4.38 ± 0.01 ab
Flood cultivation and High bed and low ditch planting system	rice	high-bed and low-ditch intercropping	6.53 ± 0.06 b
		flooding monoculture	6.69 ± 0.04 ab
	*Solanum nigrum* L.	high-bed and low-ditch intercropping	6.75 ± 0.04 a

Note: The values represent the means (±standard error) of data obtained in the experiment (*n* = 3); different letters in the same column indicate significant differences between treatments (*p* < 0.05).

## Data Availability

Data are contained within the article.
